# Diffeomorphic Registration With Intensity Transformation and Missing Data: Application to 3D Digital Pathology of Alzheimer's Disease

**DOI:** 10.3389/fnins.2020.00052

**Published:** 2020-02-11

**Authors:** Daniel Tward, Timothy Brown, Yusuke Kageyama, Jaymin Patel, Zhipeng Hou, Susumu Mori, Marilyn Albert, Juan Troncoso, Michael Miller

**Affiliations:** ^1^Department of Biomedical Engineering, Johns Hopkins University, Baltimore, MD, United States; ^2^Center for Imaging Science, Johns Hopkins University, Baltimore, MD, United States; ^3^Department of Pathology, Johns Hopkins University School of Medicine, Baltimore, MD, United States; ^4^Department of Radiology, Johns Hopkins University School of Medicine, Baltimore, MD, United States; ^5^Department of Neurology, Johns Hopkins University School of Medicine, Baltimore, MD, United States

**Keywords:** neuroimaging, digital pathology, histology, brain mapping, image registration, missing data

## Abstract

This paper examines the problem of diffeomorphic image registration in the presence of differing image intensity profiles and sparsely sampled, missing, or damaged tissue. Our motivation comes from the problem of aligning 3D brain MRI with 100-micron isotropic resolution to histology sections at 1 × 1 × 1,000-micron resolution with multiple varying stains. We pose registration as a penalized Bayesian estimation, exploiting statistical models of image formation where the target images are modeled as sparse and noisy observations of the atlas. In this injective setting, there is no assumption of symmetry between atlas and target. Cross-modality image matching is achieved by jointly estimating polynomial transformations of the atlas intensity. Missing data is accommodated via a multiple atlas selection procedure where several atlas images may be of homogeneous intensity and correspond to “background” or “artifact.” The two concepts are combined within an Expectation-Maximization algorithm, where atlas selection posteriors and deformation parameters are updated iteratively and polynomial coefficients are computed in closed form. We validate our method with simulated images, examples from neuropathology, and a standard benchmarking dataset. Finally, we apply it to reconstructing digital pathology and MRI in standard atlas coordinates. By using a standard convolutional neural network to detect tau tangles in histology slices, this registration method enabled us to quantify the 3D density distribution of tauopathy throughout the medial temporal lobe of an Alzheimer's disease postmortem specimen.

## 1. Introduction

High-throughput neuroinformatics and image analysis are emerging in neuroscience (Miller et al., 2013a; Mori et al., 2013). Atlas-based image analysis plays a key role, as it enables information encoded by millions of independent voxel measurements to be reconstructed in ontologies of the roughly 100 evolutionarily stable structures. At the 1-millimeter scale, there are many atlases, including Tailarach coordinates (Talairach and Szikla, 1980) and the Montreal Neurological Institute (MNI) (Evans et al., 1993) and Mori's diffusion tensor imaging (DTI) white matter (Mori et al., 2005) atlases, which define the locations of neuroanatomical structures as well as important structural and functional properties such as volume, shape, blood oxygen level-dependent (BOLD) signals, etc. At the millimeter scale, there have been several approaches for mapping onto atlases (Miller et al., 1993; Grenander and Miller, 1998; Toga and Thompson, 2001; Thompson and Toga, 2002; Ashburner and Friston, 2007; Ashburner, 2009), atlas estimation (Durrleman et al., 2008; Vialard et al., 2011), and applications in white matter (Zhang et al., 2010b) or even cardiac imaging (Zhang et al., 2010a; Ardekani et al., 2012). At micron and meso-scales, there are several atlases including Mori's and the Allen brain atlas (Chuang et al., 2011; Sunkin et al., 2013) with their associated region and cell-types. Many of the brain mapping algorithms have been extended to micron scales, such as for CLARITY (Chung et al., 2013; Epp et al., 2015; Kutten et al., 2017).

However, many of the dense brain mapping methods have been based on high-quality datasets, in which image collection is highly controlled and similar image modalities are being registered. In the work described here, we focus on an application that is ubiquitous in digital pathology, where micron-thick tissue slices are prone to damage and are sparse, implying large numbers of missing sections. As well, many stains are often used, which results in a multitude of contrast variations between imaged sections. In the work proposed here, we develop a generative probabilistic model that accounts for differences in shape, contrast, and sparsity associated with censoring of data samples using the random orbit model of Computational Anatomy (Grenander and Miller, 1998, 2007), in which the space of histological images is an injection into the orbit of exemplar templates under both smooth diffeomorphic coordinate transformation and image contrast transformation. The models for coordinate transformations are taken from diffeomorphometry (Miller et al., 2014, 2018). We pose registration as a penalized Bayesian estimation, exploiting statistical models of image formation where the target images are modeled as sparse and noisy observations of the atlas. The correspondence between histology images and 3D atlases is an injection, not an invertible diffeomorphism. The penalized Bayes estimator we derive applies transformations only to the atlas, removing the assumption of symmetry between atlas and target. We accommodate the space of differing contrasts associated with different histological stainings (such as tau, amyloid, myelin, Nissl, and fluorescence) by modeling the space of contrasts via polynomial functions of the atlas. Application of higher-order polynomials describes non-monotone transformations that swap the order of intensities, with first-order monotone polynomials reducing to affine transformations given by the normalized cross-correlation cost function. To accommodate effects such as folding and distortion, we include additional homogeneous atlases and model each pixel in an observed image as a realization of one transformed atlas from this family. The atlas label at each pixel is interpreted as missing data, with the conditional mean computed to estimate them using the Expectation-Maximization algorithm (Dempster et al., 1977).

Alternatives to the approach presented here for missing data have included masking of the image similarity objective function (Brett et al., 2001; Stefanescu et al., 2004) or filling with a specific image intensity or texture (Sdika and Pelletier, 2009), as well as explicit joint estimation for missing data, such as that associated with excised tissue (Nithiananthan et al., 2012) or occlusion by lesions (Yoo et al., 2002; Avants et al., 2014), tuberculosis (Vidal et al., 2009), or tumor (Zacharaki et al., 2008).

Registration between different modalities in the presence of missing data is more challenging because image abnormalities are difficult to detect when their expected contrast is unknown. Our approach of attempting to solve the Bayes problem, removing the nuisance variables as much as possible via the Expectation-Maximization algorithm, is more similar to (Periaswamy and Farid, 2006; Chitphakdithai and Duncan, 2010a). Objective functions for multimodality image similarity, such as normalized cross-correlation (Avants et al., 2006; Wu and Tang, 2018), mutual information (Mattes et al., 2003; Pluim et al., 2003), or local structural information (Heinrich et al., 2012; Wachinger and Navab, 2012; Bashiri et al., 2019) do not easily extend to an expectation maximization setting because they do not correspond to a data log-likelihood. Our framework applies to general image registration problems and differs from the methods surveyed above in several important respects. The method proposed here supports large deformations within the computational anatomy random orbit model, unlike (Periaswamy and Farid, 2006; Bashiri et al., 2019), which considers low-dimensional scale rotation and translation dimensions. Secondly, we introduce the many dimensions of polynomials to overcome the nonmonotonicity of the transformation for crossing modalities for histology jointly with deformation, unlike example (Periaswamy and Farid, 2006), which considers multimodality registration and missing tissue separately. Finally, by introducing the EM algorithm, we approximate the full Bayes problem of removing the nuisance dimensions of missing and/or distorted tissue, thereby in part benefiting from the reduced dimensional setting of EM as a solution. The potential impact of these methods has been recognized by the community, and a recent review by Pichat et al. (2018) summarizes work in this area over the past several decades. Artifacts addressed by our method (folds, tears, cracks, and holes) are specifically described as challenges but are not modeled by the registration approaches surveyed. In the survey, these factors are handled by techniques such as manual labeling of artifact regions or modified image similarity metrics such as sum of absolute deviation that avoid strongly penalizing outliers.

We apply these techniques to an important application in Alzheimer's disease (AD), computing the 3D density of tau neurofibrillary tangles, a key pathologic feature of AD (Mirra et al., 1997). Since Braak and Braak's original 1991 staging of Alzheimer's (Braak et al., 2006), it has become clear that one of the earliest locations of degeneration associated with tauopathy is the lateral boundary of the entorhinal cortex, subsequently spreading throughout the medial temporal lobe. In our previous works (Miller et al., 2013b, 2015; Younes et al., 2014, 2019; Tward et al., 2017; Kulason et al., 2019) based on 1.5 T Magnetic Resonance Imaging (MRI), we demonstrated that there is evidence of consistent atrophy, as manifested by thickness and volume measures. This appears earliest in the entorhinal cortex, with subsequent statistically significant changes seen in the amygdala and hippocampus. Aligning histological sections (stained for tau, amyloid, and myelin) with the dense 3D coordinates of individual brains offers the opportunity to associate the histological changes to pathological tau observed post mortem with structural changes observed clinically. We detect tau tangles using a standard deep convolutional neural network, where 56 × 56 pixel patches are used to classify the center of the patch. The network is applied to the whole image through overlapping patches in a scanning window approach, shifting by 1 pixel at a time. As this is a large-scale task but not a particularly challenging one, this architecture was taken from an official TensorFlow tutorial, and our work here is not intended to advance the state of the art in neural network design.

At the time of manuscript preparation, code to perform 3D registration with this algorithm is available on GitHub (https://github.com/dtward/image_lddmm_tensorflow), implemented in Python^1^ with high-performance computing handled using TensorFlow^2^ and available on any operating system that runs Python and TensorFlow. This repository includes example usage in the form of Jupyter notebooks, with figures and explanations of each step, for several datasets covering human, mouse, and rat. The implementation of our algorithm lddmm.py includes descriptions of each step in the form of comments. Our most up-to-date registration code will be described at^3^.

Our central contribution is to build a registration algorithm by extending the computational anatomy random orbit model in two ways: first, by including nonmonotonic contrast variation in addition to geometric variation, and second, by simultaneously accommodating artifacts or missing tissue through an EM algorithm. Our paper is structured as follows. In section 2, we describe our registration algorithm in three steps: (i) reviewing a traditional approach, (ii) adding contrast variation, and (iii) adding missing data. In section 3, we describe five experiments used to illustrate and validate our method: (i) with a simulated image, (ii) examining our first and second contribution in isolation and together using histology data, (iii) quantitatively demonstrating that our accuracy performance is similar to state-of-the-art alternatives using the CIMA benchmarking dataset described in Borovec et al. (2018), which includes contrast variation but not missing tissue, (iv) quantifying our accuracy via Dice overlap in a missing data setting, and (v) applying our method to Alzheimer's disease research, detecting tau tangles in 2D histology and registering them to the standard 3D coordinate system of the Mai Paxinos Voss atlas (Mai and Paxinos, 2011). In section 4, we present the results of these five experiments. Finally, in section 5, we discuss the implications of this work.

## 2. Methods

In this section, we first review the optimality conditions for the original LDDMM image registration algorithm. We then show our first contribution, how they are modified to include contrast variation, and our second contribution, how they are modified to simultaneously include missing data. In each case, we present three sets of equations: (i) a generative model, (ii) an objective function to be minimized, and (iii) optimality conditions. We conclude this section by summarizing our registration algorithm.

### 2.1. Background: LDDMM and the Geometric Transformation Problem

Our generative model for the space of observed images builds upon the deformable templates of (Grenander, 1994; Grenander and Miller, 2007). In the original setting, observed images are spatial transformations of grayscale atlases *I*:*X* ⊂ ℝ^3^ → ℝ.

Geometric differences are modeled as diffeomorphisms, φ ∈ *Diff*:*X* → *X*, which are generated from flows of smooth velocity fields *v*_*t*_, *t* ∈ [0, 1] as in (Grenander and Miller, 1998).

(1) $$\begin{array}{l} {\dot{\phi }}_{t}=v_{t}\left(\phi_{t}\right),\;\phi_{0}=id,\;\varphi =\phi_{1}, \end{array}$$

Observed images *J* are modeled as conditionally Gaussian random fields, with the mean given by the deformed template, and the addition of white noise:

$$\begin{array}{l} J\left(\cdot \right)=I\left(\varphi^{-1}\left(\cdot \right)\right)+\text{noise}\left(\cdot \right) \end{array}$$

With noise variance $\sigma_{M}^{2}$, this gives a log-likelihood function only of the parameters

$$\begin{array}{l} -\ell (J;\varphi )\;=\frac{1}{2\sigma_{M}^{2}}\|J-I\circ \varphi^{-1}{\|}_{L_{2}}^{2}\;. \end{array}$$

Because velocity fields are not finite-dimensional, they are regularized by introducing a Sobolev norm as a running penalty, weighted by the parameter $\sigma_{R}^{2}$, giving an objective function

$$\begin{array}{l} E(\varphi )=\frac{1}{2\sigma^{2}}\int_{0}^{1}\int_{X}Av_{t}\cdot \;v_{t}dxdt+\frac{1}{2\sigma_{M}^{2}}\|J-I\circ \varphi^{-1}{\|}_{L_{2}}^{2} \end{array}$$

Here, *A* is a differential operator of the form (*id* − *a*^2^Δ)^4^, where *a* is a smoothness length scale.

This problem was solved in Beg et al. (2005) with necessary conditions

(2) $$\begin{array}{l} \nabla E=\frac{1}{\sigma_{R}^{2}}Av_{t}-\frac{1}{\sigma_{M}^{2}}\nabla \left(I\left(\varphi_{t}^{-1}\right)\right)\left(I\left(\varphi_{t}^{-1}\right)-J\left(\varphi_{1t}^{-1}\right)\right)|D\varphi_{1t}^{-1}|=0 \end{array}$$

where $\varphi_{1t}=\varphi_{t}\circ \varphi_{1}^{-1}$ and |*Dφ*| is determinant of the Jacobian matrix.

### 2.2. Polynomial, Non-monotonic Mappings, and the Contrast Transformation Problem

Our first contribution is to expand the generative model to allow observed images to have a different non-monotonically transformed contrast profile from the atlas. We consider atlases *I*:*X* → ℝ^*N*^ and observed images *J*:*X* → ℝ^*M*^. These may be single-valued (*N* = 1), such as a T1 MRI, or multi-valued, such as red-green-blue (RGB, *N* = 3). We model this change in contrast by a polynomial function of specified degree with coefficients θ, denoted *F*_θ_.

The generative model describes *J* as a conditionally Gaussian *M* vector field with each component of noise independent,

$$\begin{array}{l} J\left(\cdot \right)=F_{\theta }\left[I\left(\varphi^{-1}\left(\cdot \right)\right)\right]+\text{noise}\left(\cdot \right). \end{array}$$

Our objective function is

(3) $$\begin{array}{l} \begin{array}{l} E(\varphi ,\theta )=\frac{1}{2\sigma_{R}^{2}}\int_{0}^{1}\int_{X}Av_{t}\cdot \;v_{t}dxdt+\frac{1}{2\sigma_{M}^{2}}\|J-F_{\theta }(I\circ \varphi^{-1}){\|}_{L_{2}}^{2} \end{array} \end{array}$$

where the *L*_2_ norm sums over each component of vectors in ℝ^*M*^. Our two necessary conditions are

(4a) $$\begin{array}{l} \nabla_{v}E=\frac{1}{\sigma_{R}^{2}}Av_{t}-\frac{1}{\sigma_{M}^{2}}\nabla [F_{\theta }(I(\varphi_{t}^{-1}))](F_{\theta }(I(\varphi_{t}^{-1}))\\ \;-J(\varphi_{1t}^{-1}))|D\varphi_{1t}^{-1}|=0 \end{array}$$

(4b) $$\begin{array}{l} \nabla_{\theta }E=\int_{X}F_{\theta }(I\circ \varphi_{1}^{-1}(x))\cdot \frac{d}{d\theta }F_{\theta }(I\circ \varphi_{1}^{-1}(x))dx\\ \;-\int_{X}J(x)\cdot \frac{d}{d\theta }F_{\theta }(I\circ \varphi_{1}^{-1}(x))dx=0 \end{array}$$

The first equation (Equation 4a) is a minor modification to Beg's algorithm (2), and the second (Equation 4b) solves for θ as the solution to a least-squares problem. Generally, *F*_θ_ is nonmonotic polynomial, but when it is an affine transformation, this algorithm is equivalent to minimizing a normalized cross-correlation loss function (see Appendix 1 in Supplementary Material).

### 2.3. EM Algorithm and the Missing Data Problem

Our second contribution is to expand the generative model to allow observed images to have a different identity at each pixel. For example, pixels showing torn or folded tissue may be labeled as “background” or “artifact” (respectively) and not correspond to any location in the atlas through the transformation φ. In this setting, missing data takes the form of per-pixel labels that indicate which member of a family of atlases the intensity at that pixel comes from. Thus, the missing tissue (due to a tear or a fold) would be modeled by a “background” atlas, and corrupted pixels due to a streak or a smudge would be modeled by a “artifact” atlas. We approach the problem of missing or censored data using the Expectation-Maximization algorithm (Dempster et al., 1977).

Our new generative model describes *J* with multiple possible means and variances at each pixel *i*

(5) $$\begin{array}{l} J_{i}=\{\begin{array}{ll} F_{\theta }{\left[I(\varphi^{-1})\right]}_{i}+{\text{noise}}_{i}^{M} & \text{if voxel }i\text{ is tissue}\\ \mu_{A}+{\text{noise}}_{i}^{A} & \text{if voxel }i\text{ is artifact}\\ \mu_{B}+{\text{noise}}_{i}^{B} & \text{if voxel }i\text{ is background} \end{array} \end{array}$$

the noise components have variance $\sigma_{M}^{2}$, $\sigma_{A}^{2}$, and $\sigma_{B}^{2}$.

Our EM algorithm for jointly estimating the deformation, contrast transformation, and class of each voxel is derived in Appendix 2 in Supplementary Material. At each M step, we update our transformation parameters by minimizing the objective function

(6) $$\begin{array}{l} E(\varphi ,\theta )=\frac{1}{2\sigma_{R}^{2}}\int_{0}^{1}\int_{X}Av_{t}\cdot \;v_{t}dxdt\\ \;+\frac{1}{2\sigma_{M}^{2}}\sum_{i}|J_{i}-F_{\theta }{\left[I\circ \varphi^{-1}\right]}_{i}){|}^{2}\pi_{i}^{M} \end{array}$$

where $\pi_{i}^{M}$ is the posterior probability that voxel *i* corresponds to our atlas image (rather than background or artifact). Our necessary conditions are

(7a) $$\begin{array}{l} \nabla_{v}E=\frac{1}{\sigma_{R}^{2}}Av_{t}-\frac{1}{\sigma_{M}^{2}}\nabla [F_{\theta }(I(\varphi_{t}^{-1}))](F_{\theta }(I(\varphi_{t}^{-1}))\\ \;-J(\varphi_{1t}^{-1}))|D\varphi_{1t}^{-1}|\pi^{A}\circ \varphi_{1t}^{-1}=0 \end{array}$$

(7b) $$\begin{array}{l} \nabla_{\theta }E={\sum_{i}F_{\theta }\left[I\circ \varphi_{1}^{-1}(x)\right]}_{i}\cdot \frac{d}{d\theta }F_{\theta }{\left[I\circ \varphi_{1}^{-1}(x)\right]}_{i}\pi_{i}^{M}\\ \;-\sum_{i}J_{i}\cdot \frac{d}{d\theta }F_{\theta }{\left[I\circ \varphi_{1}^{-1}(x)\right]}_{i}\pi_{i}^{M}=0 \end{array}$$

(7c) $$\begin{array}{l} \mu_{A}=\sum_{i}J_{i}\pi_{i}^{A}/\sum_{i}\pi_{i}^{A},\;\mu_{B}=\sum J_{i}\pi_{i}^{B}/\sum_{i}\pi_{i}^{B} \end{array}$$

Here, π^*M*^ is interpreted as a continuous function using linear interpolation, and $\pi_{i}^{A},\pi_{i}^{B}$ are the posterior probability that voxel *i* corresponds to an artifact or background (respectively). The first equation (Equation 7a) differs from Equation (4a) only by the factor π^*M*^, and the second equation (Equation 7b) differs from Equation (4b) by the factor π^*M*^. For technical reasons, we use discrete notation in Equations (7a) and (7b).

### 2.4. Optimization Algorithm

Our resulting EM-LDDMM optimization algorithm is summarized in Algorithm 1. See the Appendix 2 in Supplementary Material for a proof that this is an EM algorithm and thus monotonically increasing in likelihood.

**Algorithm 1:** EM-LDDMM for image registration with intensity transformations and missing data. Below, we use two possibilities for missing data, artifact, or background.

**Input data**: Atlas image *I* and target image *J*.
**Input parameters**: σ_*M*_, σ_*A*_, σ_*B*_ (weights in cost function), *a* (length scale for transformation smoothness), *O* (order of polynomial for contrast mapping).
**Initialization**: *v*_*t*_ = 0 (zero velocity field for identity transform), θ = (0, 1, …) polynomial coefficients for identity transform, μ_*A*_, μ_*B*_ artifact and background intensities.
**Optimization**: Repeat until convergence. **E step:** Compute π_*a*_ for matching, artifact, and background using Equation (9). **M step:** Repeat until convergence. **Forward pass**: Compute $\varphi_{t}^{-1}$ from *v*_*t*_ for *t* ∈ [0, 1] using Equation (1), and compute $F_{\theta }\left[I\left(\varphi_{1}^{-1}\right)\right]$. **Objective**: Evaluate the objective function for *v*_*t*_, $F_{\theta }\left[I\left(\varphi_{1}^{-1}\right)\right]$ and *J* using Equation (6). **Backward pass**: Compute θ, μ_*A*_, μ_*B*_ by weighted least-squares using Equation (7b) and Equation (7c). Compute φ_*t*_ from *v*_*t*_ using Equation (1) and update *v*_*t*_ by gradient descent using Equation (7a).
**Output**: Forward and inverse transforms $\varphi_{1},\varphi_{1}^{-1}$ (optionally output other parameters of interest).

## 3. Experimental Setting and Material

In this section, we describe the imaging data acquired as part of our Alzheimer's disease study and the five registration experiments used to illustrate and validate our method.

### 3.1. Post-mortem Imaging

Preparation and scanning of brain tissue were performed by the neuropathological team at the Johns Hopkins Brain Resource Center (BRC) and the laboratory of Dr. Susumu Mori. The specimen was a 1,290 g brain from a 93-year-old male, with a clinical diagnosis of Alzheimer's disease dementia. The autopsy diagnoses included: Alzheimer's disease neuropathologic change, high level (A3,B3, C2) (Hyman et al., 2012); CERAD neuritic plaque score B (Mirra et al., 1991); neurofibrillary Braak stage VI/VI (Braak et al., 2006); subacute infarcts frontal, temporal, and basal ganglia; old infarct of pons; with clinical-pathological comment: “Mixed dementia, AD, and vascular. The AD component appears to predominate.” The fixed brain tissue was divided into six coronal blocks of the temporal lobe that contain the entorhinal cortex, the hippocampus, and the amygdala. The orientations of the blocks correspond as closely as possible to the coordinate system of the Mai Atlas. Each block of brain tissue was scanned with a high-field 11.7T MRI scanner.

The nuclear magnetic resonance (NMR) sequence was based on a 3D multiple echo sequence (Mori and Van Zijl, 1998; Xue et al., 2001), with four echoes acquired for each excitation. The diffusion-weighted images were acquired with a field of view of typically 40 × 30 × 16 mm and an imaging matrix of 160 × 120 × 64, which was zero-filled to 320 × 240 × 128 after the spectral data were apodized by a 10% trapezoidal function. The pixel size was native 250-micron isotropic. Eight diffusion-weighted images were acquired with different diffusion gradient directions, with *b*-values in the 1,200–1,700 s/*mm*^2^ range. For diffusion-weighted images, a repetition time of 0.9 s, an echo time of 37 ms, and two signal averages were used, for a total imaging time of 24 h.

**Figure 1:**
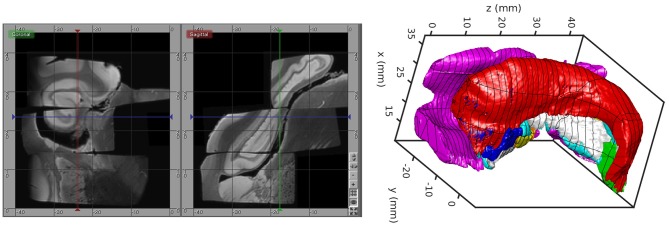
High-field MTL volume from MR. **(Left)** MR images of tissue blocks aligned in 3D. **(Right)** Surface rendering of manual segmentations with Mai Atlas coordinate system superimposed.

The MRI scanning procedure resulted in several distinct images that must themselves be aligned after imaging. For this, we developed an interactive tool for visualizing and transforming each imaged block to match with the others and for rigidly positioning the aligned blocks in the Mai Atlas coordinate system. The blocks were aligned by manually adjusting rotations and translations by small amounts until they could not be improved. The alignment was calculated by a biomedical engineer with feedback provided by a neuroanatomist. An example of our images and their alignment is shown on the left in Figure 1. The aligned blocks were labeled by a neuroanatomist for relevant medial temporal lobe structures [entorhinal cortex, subiculum, Cornu Ammonis (CA) fields, compartments of dentate gyrus, alveus]. These labels are shown as a surface reconstruction on the right in Figure 1. The superimposed lines correspond to the rostral-caudal pages of the Mai-Paxinos-Voss atlas (z-axis), with their 1 cm grid lines along the x-y axes of each page. Our definition of the entorhinal cortex (magenta) includes the medial bank of the collateral sulcus. This sulcal region (Krimer et al., 1997), also referred to as the trans entorhinal cortex, corresponds to the earliest location of AD pathology accumulation visible at autopsy (Braak and Braak, 1991). Atrophy in this region has been detected at the population level in subjects with mild cognitive impairment (Albert et al., 2011) before other changes are visible (Tward et al., 2017).

After the tissue blocks underwent high-field imaging, they were sectioned for histological examination. The tissue was sectioned at 200 μm intervals: 20 slices of 10 μm thickness and 5 slices of 40 μm thickness. The thin-sliced sections are prepared with stains focused on AD pathology: Nissl, silver (Hirano method), Luxol fast blue (LFB) for myelin, and immunostained for Aβ (mab 6E10) and tau (PHF1). Several examples of our tau, amyloid, and myelin stained sections are shown in the results section (**Figures 3**–**11**). For future analysis, these stains are complemented by additional stains to examine the tissue for related comorbidities and other neurodegenerative disorders, such as Lewy body disease and frontotemporal dementia, including immunostains for α-synuclein, ubiquitin, TDP-43, GFAP (astrocytes), Iba1 and CD68 (microglia), collagen IV (blood vessels), and reelin (entorhinal cortex layer II neuronal protein) 13. The thick-sliced sections were used for quantitative cell and neuron counts and density studies of dendritic and synaptic markers.

### 3.2. Image Mapping Experiments

Below, we present several 2D experiments to demonstrate the applicability and verify the validity of our method. In each case, linear and deformable registration is performed simultaneously using gradient descent, and deformable registration is implemented using LDDMM (Equation 4a) or weighted LDDMM in an EM algorithm (Equation 7a). In our first experiment, we provide an illustrative example using a pair of simulated images that contain contrast variation, missing tissue, and artifacts.

In our second experiment, we use example histology images for a qualitative evaluation of the different components of our algorithm: contrast changes only, missing tissue only, and both together. These are approximately 1-micron resolution, with typical images about 20,000 × 10,000 pixels. For registration purposes, they are downsampled by averaging over a 32 × 32 neighborhood.

In our third experiment, we demonstrate the accuracy of our algorithm using the benchmarking dataset described in (Borovec et al., 2018). We use all seven lung tissue slides corresponding to 140 registrations between pairs of stains. While this dataset does not correspond exactly to our desired application in neuroimaging, it provides a great opportunity to calculate accuracy through landmark target registration error, which was defined in the above paper as mean landmark distance error divided by the diagonal of the image, and to compare to state-of-the-art methods. In particular, this dataset allows us to study the contrast variation component of our algorithm but not the missing data component. Using the software package described in the paper^4^, we were able to compare our registration results to alternative state-of-the-art methods, using configuration settings chosen by their authors specifically for this dataset. We sought to compare to ANTs because it scored first or second place in terms of robustness and accuracy and has been used in several neuropathology applications, including Adler et al. (2014) and bUnwarpJ because the majority of our colleagues working in microscopy use this software. We also compared to the first and second place winners of the related ANHIR challenge presented at ISBI 2019^5^, Histokat and Historeg (respectively). To give further context, we also compare to results with no registration and results with the best possible affine registration (which uses information not available to the other methods).

In our fourth experiment, we demonstrate the accuracy of our algorithm with both contrast variation and missing data and compare to the partial data local affine method of Periaswamy and Farid (2006), which has 2D source code available^6^. Using a DTI atlas set from MRICloud^7^, we performed a mapping experiment from b0 images to trace images, between each pair of subjects in the atlas set, for a total of 56 maps. We chose the axial slice where lateral ventricles appeared the largest and calculated mean and standard deviation of Dice overlap of gray matter, white matter, and lateral ventricle. We registered whole brains to whole brains as a baseline, and, to examine missing data, we registered whole brains to hemispheres.

Our fifth and final example is to demonstrate an important application in a 3D histology pipeline, quantifying the 3D distribution of tau tangles in Alzheimer's disease. Here, we work with a sequence of transformations between 3D post-mortem MRI and 2D histology slices. The sequence consists of 3D shape change (deformation and rigid positioning), adjusting for scaling (slice spacing and pixel size), position on the microscope slide (2D rigid motion), and variation in contrast (cubic intensity transformation). Rigid, scale, and deformation parameters are all jointly optimized using gradient descent. Since these transformations are combined by composition, gradients can be calculated using straightforward backpropagation (chain rule). Deformable registration uses a relatively small gradient descent step size, allowing linear transformations to be close to optimal at all times.

This pipeline quantifies the distribution of tau tangles in each 2D slice using a convolutional neural network in TensorFlow (Tward et al., 2018). Input data is 56 × 56 regions of interest, and the network architecture is summarized in Table 1. The center pixel of each region is classified as belonging to one of three classes: “tau tangles,” “other tissue,” or “background.” A total of 2,391 training examples were identified manually by randomly choosing regions of interest and mouse-clicking in the center of tau tangles or on other tissue or background, with 8.3% positive, and neural network weights were trained using the Adam optimizer (Kingma and Ba, 2014). Every pixel in our histology data was classified using a sliding window approach as in Ciresan et al. (2012). After mapping this data into the coordinates of the Mai atlas, we report the total area of tau tangles within the entorhinal cortex, subiculum, and CA1-3 for each atlas page. To place these numbers in context, we also report the total area of these anatomical regions (which may be affected by missing tissue) and the fraction of this area covered by tau tangles.

**Table 1:** Neural network architecture.

**Layer**	**Input shape**	**Parameters**
5 × 5 convolution 1	56 × 56 × 3	5 × 5 × 3 × 16 + 16 = 1, 216
ReLu 1	56 × 56 × 16	0
Max Pool 1	56 × 56 × 16	0
5 × 5 convolution 2	28 × 28 × 16	5 × 5 × 16 × 32 + 32 = 12, 832
ReLu 2	28 × 28 × 32	0
Max Pool 2	28 × 28 × 32	0
5 × 5 convolution 3	14 × 14 × 64	5 × 5 × 32 × 64 + 64 = 51, 264
ReLu 3	14 × 14 × 64	0
Max Pool 3	14 × 14 × 64	0
Flatten	7 × 7 × 64	0
Fully connected	3, 136	3, 136 × 1, 024 + 1, 024 = 3, 212, 288
ReLu	1, 024	0
Dropout	1, 024	Probability 0.5
Fully connected	1, 024	1, 024 × 3 + 3 = 3, 075
Cross entropy loss	3	0

## 4. Results

In this section, we present results and comments for each of our five image-registration experiments. In section 4.3, we particularly highlight the difference between our asymmetric generative statistical model and popular symmetric alternatives.

### 4.1. Mapping Simulated Images With Artifact and Missing Tissue

**Figure 2:**
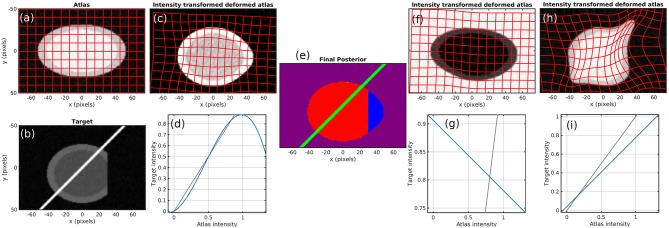
Simulation results. **(a)** atlas and **(b)** target. **(c–e)** Show results using our method, with the **(c)** transformed atlas image, **(d)** nonmonotic cubic contrast transform *F*_θ_, and **(e)** final posterior probability of atlas labels at algorithm convergence. **(f,g)** Show similar results for a fixed binary mask, and **(h,i)** show them for no mask. The identity line for contrast transforms is shown in gray. Because **(e)** does not render well in grayscale, we have presented it as three grayscale images in our Supplementary Material.

To demonstrate the method, we start with simulated images. Figure 2 shows the atlas (Figure 2a) and target (Figure 2b). Contrast is chosen so that the atlas appears like a T1 MR brain image (darker gray matter and brighter white matter) and the target appears like a T2 MR brain image. The target also contains a bright streak artifact and missing tissue. Specifically, the atlas background has intensity 0, gray matter 1, and white matter 1.25. The target background has intensity 0, gray matter 0.9, white matter 0.675, and artifact 5. Both images have additive white Gaussian noise with standard deviation 0.05 and are blurred with a Gaussian kernel with a standard deviation of 2/3 pixels over a 5 × 5 pixel window.

Figures 2c,d shows that a cubic intensity transformation is sufficient to permute the order of gray and white matter, allowing for accurate matching of the cortical boundary. Figure 2e shows the posterior probabilities of the three atlases, shown as components of an RGB image. The pixels are correctly classified, with the atlas image in red, artifact in green, and background in blue. Note that the dark region is magenta because “atlas image” and “background” both describe the image intensity equally well.

Figure 2 also shows the failed results that occur when using a linear contrast transform only (i.e., normalized cross-correlation), and a fixed mask (Figures 2f,g) or no mask (Figures 2h,i). Several alternative methods that use these approaches are described in section 1. With a fixed mask, the artifact is still handled appropriately. An inversion of contrast is estimated, which is appropriate within the masked region. However, missing tissue is not distinguished from normal background, so the informative “cortex/background” boundary is treated equivalently to the uninformative “cut tissue” boundary, resulting in very poor alignment. With no mask, huge distortions in shape occur as the atlas is squeezed to match the shape of the target with missing tissue and stretched to follow the bright artifact.

The number of constant-valued atlases used in addition to our template image is of importance. Because many neuroimages are approximately piecewise constant (e.g., a single intensity value for each of gray matter, white matter, and cerebrospinal fluid), using many constant atlases has the potential for overfitting. In the simulated example here, using four such atlases can describe the target image exactly by assigning a probability of zero to our non-constant atlas. This would give an undesirable solution that avoids registration entirely. In all the work shown here, we use one or two constant-valued atlases corresponding to “background” or “background and artifact.” This small number of atlases leads to good quality registration without overfitting.

### 4.2. Mapping Histology With Missing Tissue and Different Stains

**Figure 3:**
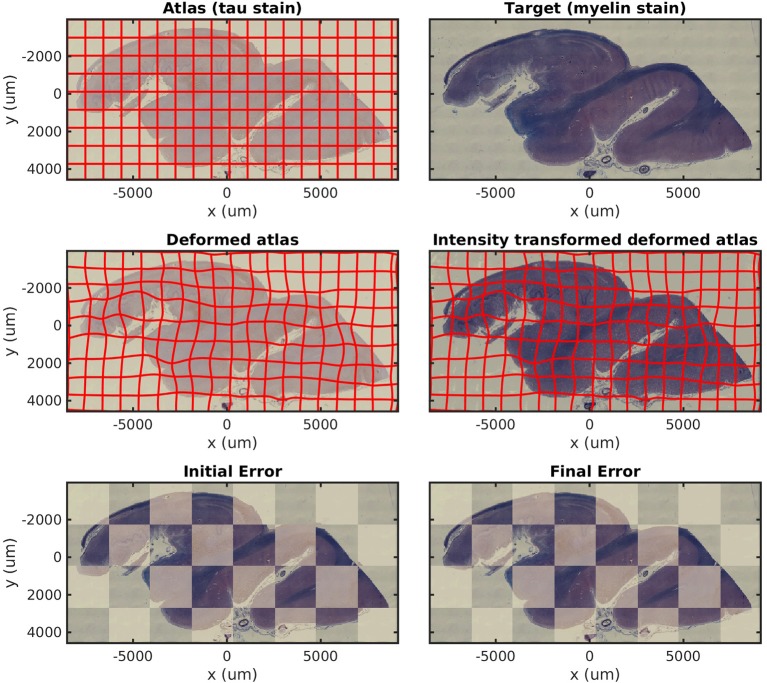
Mapping across modality from tau to myelin. A tau-stained section through the hippocampus is mapped to a neighboring myelin-stained section using a cubic polynomial intensity transform.

In Figure 3, we show results mapping a tau-stained section of the medial temporal lobe to an immediately adjacent section stained with LFB. We perform intensity transformation using a non-monotonic cubic polynomial, allowing for a swapping of brightness from gray matter (1) → white matter (2) → background (3) in tau to (2) → (1) → (3) in LFB. As a map from ℝ^3^ → ℝ^3^, this corresponds to 60 unknown parameters (one constant, three linear, six quadratic, and 10 cubic, for each of three dimensions). This example illustrates the intensity transformation component of our algorithm in isolation.

**Figure 4:**
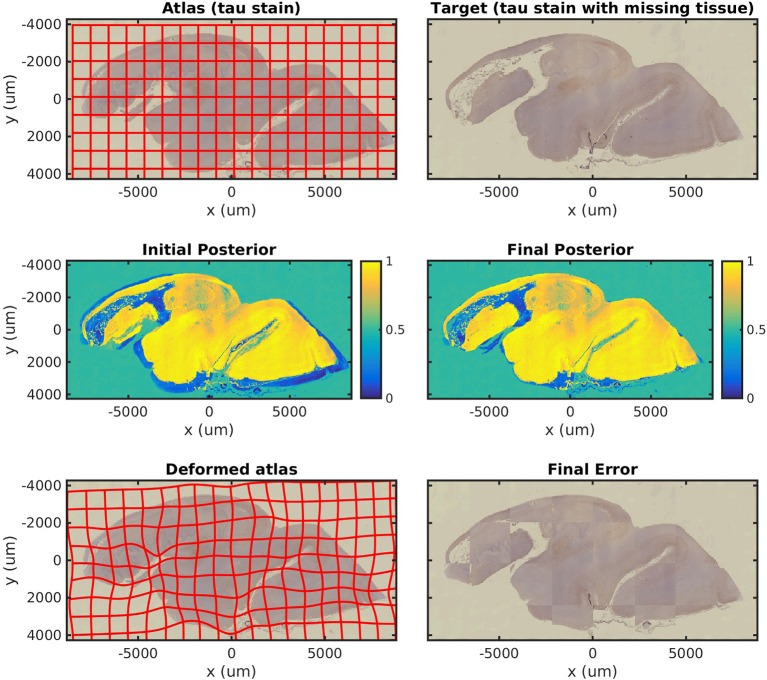
Mapping a tau section with missing tissue. A tau-stained section through the hippocampus is mapped to a neighboring damaged section. The weights show the posterior probability that a given pixel has an atlas label that corresponds to our tau-stained image, not background. High probability is shown in yellow, probability 0.5 in green, and low probability in blue.

In Figure 4, we map a tau-stained slice of medial temporal lobe to its neighbor, which has significant missing tissue due to damage. This illustrates the missing data component of our algorithm in isolation. In this experiment, two atlas labels are used: “tau-stained image” and “background.” The top right panel shows the posterior probability that each pixel corresponds to the first label at the initialization of our algorithm, and the middle right panel shows the same probability after algorithm convergence.

**Figure 5:**
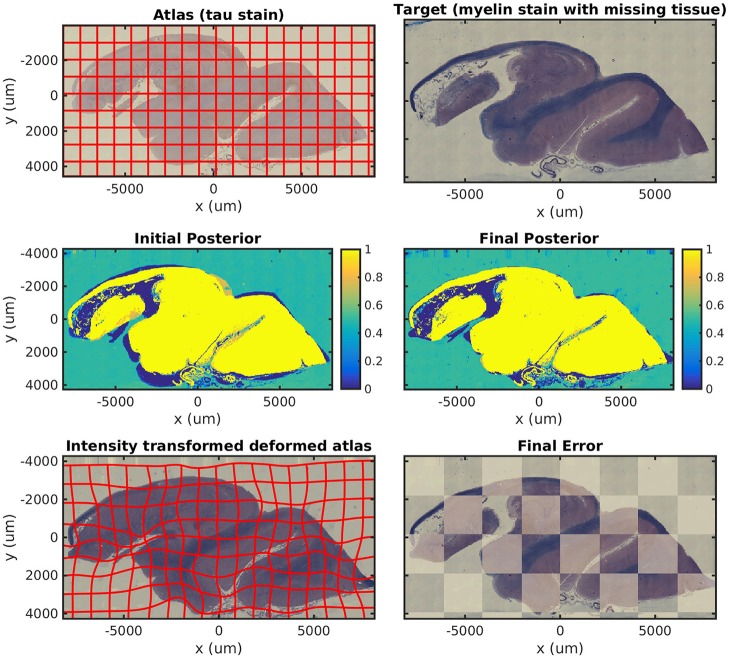
Mapping across modality from tau to myelin with missing tissue. A tau-stained section through the hippocampus is mapped to a neighboring LFB-stained damaged section. The weights show the posterior probability that a given pixel has an atlas label that corresponds to our tau-stained image, not background. High probability is shown in yellow, probability 0.5 in green, and low probability in blue.

In Figure 5, we show results mapping a tau-stained section of the medial temporal lobe to an adjacent damaged slice stained with LFB. This illustrates the intensity mapping and missing data components of our algorithm simultaneously.

### 4.3. Quantitative Analysis on Standardized Benchmark Datasets

Our statistical model of image formation removes the symmetry assumptions between atlas and target used in other methods (Christensen and Johnson, 2001; Avants et al., 2006, 2008), since the correspondence of histology images with 3D atlases is typically a non-invertible injection. The penalized Bayes estimator therefore computes deformations and variations only in the template image, which are compared to the histology targets via log-likelihood. For example, Equation (7a) only includes a gradient of the atlas image, whereas symmetric methods would require gradients of atlas and target. Because of missing tissue and sparse sampling, there is no 3D gradient in the target histology coordinates.

**Figure 6:**
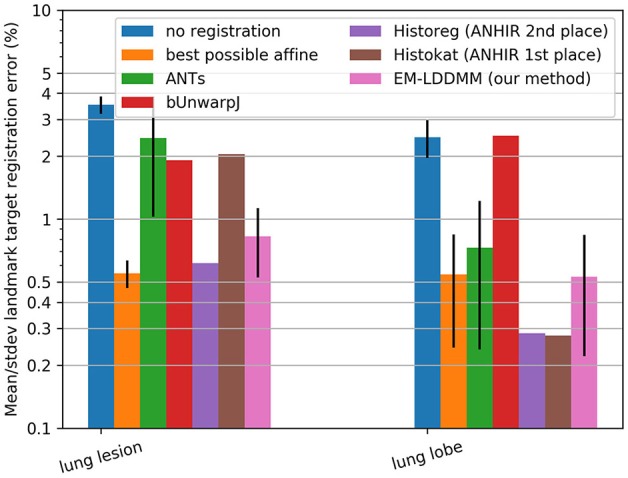
Comparison on standard datasets. Landmark target registration error on benchmarking dataset (mean, and standard deviation if available, across all datasets in each category). To give a sense of scale, we also include error without registration and with the best possible affine registration (which uses landmark location information not available to the other methods).

This suggests that if the template and target are of the same dimension and have similarly low noise, then the symmetric constraint is an excellent assumption and will perform well. Shown in Figure 6 are comparisons of our method to alternatives including the symmetric approach ANTs, showing the landmark-based accuracy between pairs of histology stains in seven datasets. The results show that our method gives performance similar to that of other state-of-the-art techniques on this well-controlled dataset without missing tissue or artifacts. We obtained mean and standard deviation results for ANTs by running it locally using provided configuration files, and downloaded others from the ANHIR challenge website from which mean but not standard deviation was available. The computation time for registering these benchmark images was on average 6.6 ± 2.1 minutes when run using a standard Python implementation (FFTs computed using NumPy and interpolation computed using scipy^8^) with no explicit GPU or multi-threading support. Our system uses two 6 core Intel Xeon processors at 3.6 GHz.

**Figure 7:**
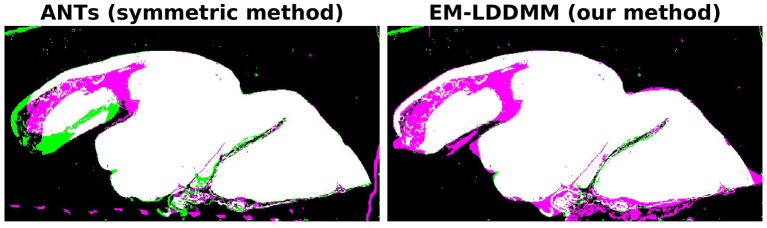
Comparison of symmetric and asymmetric methods on a histology section with missing tissue. Deformed atlas is shown in magenta, and target image is shown in green. The two are combined by summing in RGB space. Black and white colors mean background and foreground are matching, respectively. Magenta implies deformed atlas tissue is present but not target, and green the opposite. While the symmetric method shows magenta and green, our asymmetric method shows almost entirely magenta.

It is interesting to compare the symmetric and non-symmetric approaches when we move toward scenarios where the target has significant missing or damaged tissue. Our example in Figure 5 is one such case, and in Figure 7, we compare our results to the symmetric method. The figure shows overlaps between the mapped histology section and a target section that has missing tissue. The images were generated by applying binary masks and summing the two images in RGB space. Black and white colors mean background and foreground are matching, respectively. Magenta implies that deformed atlas tissue is present but not target, and green the opposite. The symmetric method results in a roughly equal amount of magenta and green, whereas our asymmetric method results in almost entirely magenta. In situations like this one, where it is true that tissue is missing, we expect our asymmetric method to provide the desired result.

### 4.4. Dice Overlap for Whole Brains and Hemispheres

**Figure 8:**
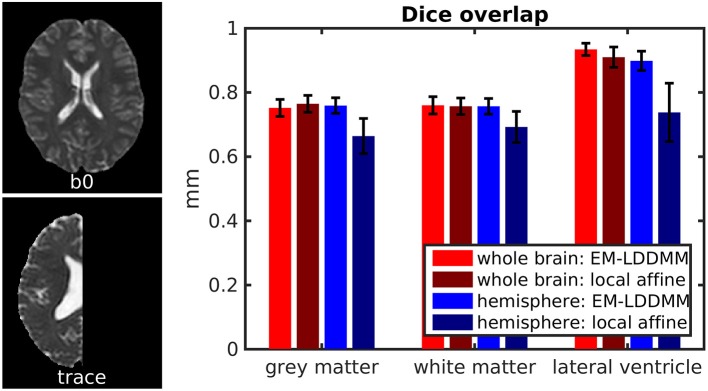
Evaluation of contrast variation and missing data using Dice overlap. We evaluate registration accuracy via Dice overlap for gray matter, white matter, and lateral ventricle. **(Left)** example b0 and trace images used, illustrating whole brain and hemisphere slices. **(Right)** Dice scores for whole-brain registration are shown in red, and whole brain to hemisphere in blue.

We show an example pair of images from our Dice overlap experiment in Figure 8 left. This illustrates the contrast differences between b0 and trace images and also illustrates our generated hemispheres. The mean and standard deviation of Dice scores across 56 mappings is shown on the right in Figure 8 for gray matter, white matter, and lateral ventricle. Both methods examined (light vs. dark colors) perform similarly for whole-brain data. Our method shows consistently higher Dice overlaps when registering whole brains to hemispheres. This difference may be explained by our method accommodating more than one type of anomalous data or explicitly using diffeomorphic transformations.

### 4.5. Mapping Histology Data to Mai Atlas Coordinates

**Figure 9:**
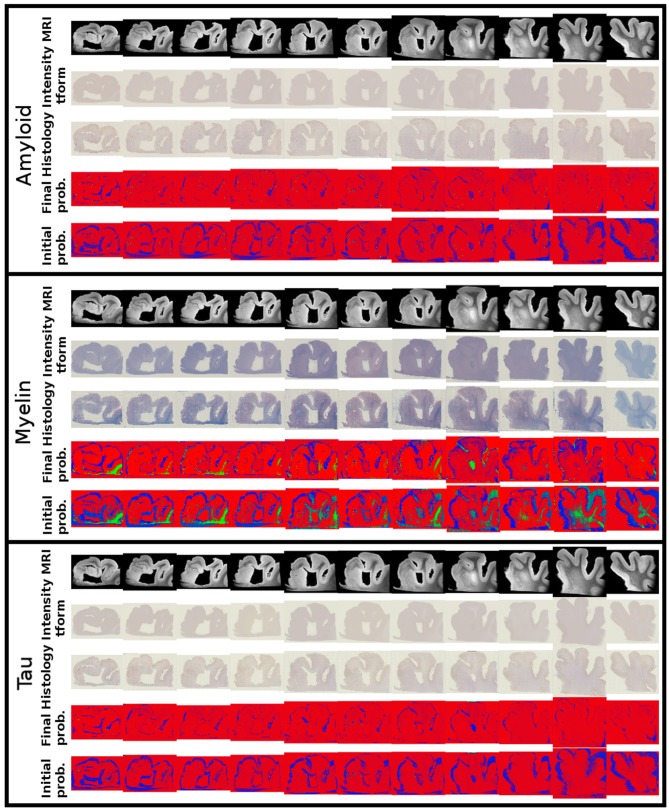
Mapping from MRI to three histology slices. Alignment between our 3D MRI and histology slices at 11 locations with three stains is shown. From top to bottom, boxes show data for amyloid, myelin, and tau stains. Within each box, from top to bottom: registered MRI, registered MRI intensity mapped to histology, histology, posterior probability of atlas labels at each pixel, posterior probability of atlas labels at each pixel at start of the algorithm. As in Figure 2, we use red: atlas image, blue: background, green: artifact.

Alignments between 3D post-mortem MRI and each of our three 2D stains are shown in Figure 9. For each stain, we show registered MRI, intensity transformed registered MRI, and histology images. We also show the posterior probability of each atlas label (“template image,” “background,” “artifact”) at the start and finish of the algorithm. Comparing the start to the finish demonstrates the quality of the alignment. Several regions where changes are not diffeomorphic, such as opening and closing of the ventricle, are labeled as artifact or missing tissue. The LFB stain, in particular, shows significant variation in contrast profiles from slice to slice, which is handled effectively by our method. All intensity transformations use a cubic polynomial for each of the red, green, and blue channels on each slice, which corresponds to 12 parameters.

**Figure 10:**
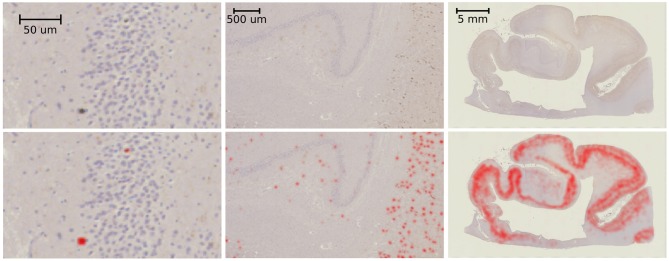
Tau-detection results. Tau-stained histology sections **(top)** with the result of our tangle detection (**bottom**, red) at three different scales, zooming out from single tangles **(left)** to the entire medial temporal lobe **(right)**.

Example results of our tau-detection algorithm, using the convolutional neural network with the architecture specified in Table 1, are shown in Figure 10. We achieve a classification accuracy of 0.996 on a test set of 500 examples that were randomly left out from the 2,391 manually classified images in our training set. The detection results are shown in Figure 10 by overlaying the image with a red color at three different scales, with individual detections at the micron level and densities at the millimeter level.

**Figure 11:**
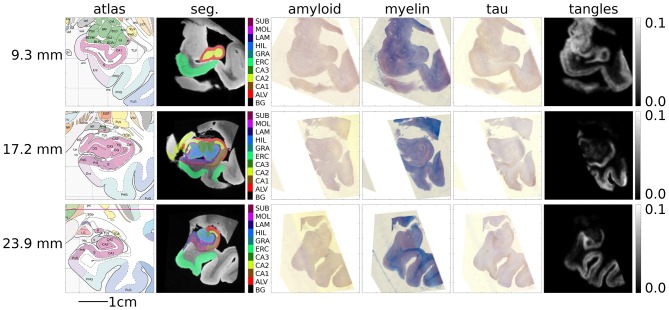
Mapping MRI sections to Mai-Paxinos sections. High-field atlas section at 9.30 mm **(top)**, 17.20 mm **(middle)**, and 23.90 mm **(bottom)** along the caudal-rostral axis of histology sections in Mai-Paxinos coordinates.

Figure 11 shows several sections of the Mai atlas along the rostral to caudal axis in millimeters. In the same coordinate system, we show our post-mortem MRI with manual segmentations superimposed and our histology stains and estimated tangle density. For visualization of this sparse data in 3D, interpolation was applied between slices. To sample at a fraction *p* between slices *I* and *J*, a symmetric LDDMM transformation was computed (Avants et al., 2008), and a weighted average of images was computed from the flow (Lee et al., 2019): $(1-p)I\circ \varphi_{t}^{-1}+pJ\circ \varphi_{1t}^{-1}$.

**Figure 12:**
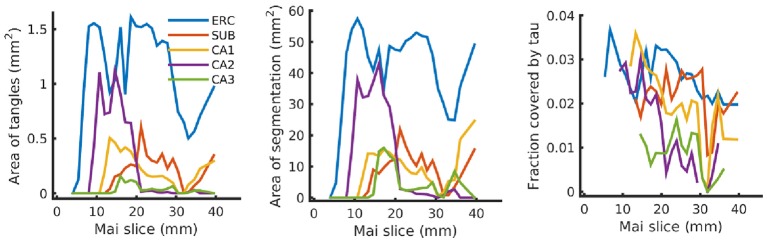
Tau tangles on each page of Mai atlas for several structures. Left shows the total area of tau tangles detected within several anatomical structures (ERC, entorhinal cortex; SUB, subiculum; CA, Cornu Ammonis). Center shows the area of these structures, and right shows the relative area of tau tangles (area covered by tau tangles divided by area of the structure).

Finally, Figure 12 shows our estimated area covered by tau tangles on each page of the Mai atlas for several structures (entorhinal cortex, subiculum, and CA fields). We observe a trend of decreasing tangle concentration in the rostral to caudal direction. Note that a 3D rendering of the segmented structures used for Figures 11, 12 is shown in Figure 1.

## 5. Conclusion

In this work, we proposed a new image-mapping method that accommodates contrast differences, missing data, and artifacts. This was achieved by formulating the imaging process as (i) an unknown shape through the action of the diffeomorphism group, (ii) an unknown change in contrast through the action of polynomial maps, and (iii) the addition of Gaussian noise. Here, (i) describes the object being imaged, and (ii) and (iii) describe the imaging process, reflecting the distinction made by Shannon between source and channel. This model allows multimodality image-matching to be formulated as a penalized likelihood problem rather than simply the maximization of an image-similarity function. This statistical model leads naturally to the formulation of an expectation-maximization algorithm that handles missing data or artifacts. We applied this algorithm to simulated images, illustrating its effectiveness for accurate mapping and classification of image pixels and its superiority over typical alternatives. For 2D histology, we qualitatively demonstrated the effectiveness of each of our two contributions, contrast mapping and tissue classification, in isolation and simultaneously. We quantitatively demonstrated that our method gives an accuracy comparable to other state-of-the-art alternatives using a benchmarking dataset that evaluates contrast changes but not missing data. Finally, we applied this technique to the challenge of reconstructing 3D volumes from histology by mapping to post-mortem MRI. In conjunction with convolutional neural networks, this allowed us to map out the 3D distribution of tau tangles in the medial temporal lobe.

Here, we demonstrated that for ℝ → ℝ affine contrast transformations, our formulation is equivalent to normalized cross-correlation. Another popular image-similarity term is mutual information, which is invariant to all invertible transformations. Our approach can accommodate these invariances if we allow for arbitrary nonparametric transformations, which can be thought of as high-degree polynomials or as linear combinations of narrow kernel functions. In this limit, a standard result of statistical prediction results in an intensity transformation given by conditional expectation, *F*(*i*) = *E*_*J*|*I* = *i*_[*J*]. This transformation results in a cost function with the same set of invariances as mutual information.

Typically, image registration has involved the balance between a regularization term and a data attachment term in optimization, which is characterized by a single parameter chosen to reflect the researcher's priorities. A limitation of our algorithm is that it requires more parameters: a variance for shape change (regularization) and variances of image noise, background noise, and artifact noise. However, rather than being chosen arbitrarily, these must be chosen carefully to reflect the physical characteristics of a random imaging model. The choice of polynomials here to describe intensity changes provides an efficient method for basis representation of the contrast or image variation. Further, as is typical of Expectation-Maximization algorithms, optimization in our setting can be slow and sensitive to initialization. We attempt to overcome this initialization issue using standard approaches in the registration community, including working from low resolution to high resolution and beginning with linear transformations and working toward deformations.

The scalability of the algorithm described is technically *O*(*N log*(*N*)), where *N* is the number of voxels in the atlas image, owing to the Fast Fourier Transform (FFT) used for applying and inverting differential operators. In practice, however, the majority of computation time is spent in linear interpolation, which is *O*(*N*). Both linear interpolation and FFTs can be very efficiently parallelized on multicore systems or graphics-processing units.

While we compared our algorithm to two state-of-the-art methods for registration with contrast differences on a standard benchmarking dataset, this was not possible for missing data methods. To place our method in context, we briefly compare to alternative modeling approaches. Quicksilver (Yang et al., 2017), a deep learning-based method, has demonstrated robustness against anomalies that are not present in the training set. While these methods show promise, our contribution is made in the setting of explicit modeling rather than implicitly learning from large datasets. Such datasets are becoming common in whole-brain MRI but are currently lacking in histology, our intended application. Other models allow transformations to become non-smooth or noninvertible at boundaries of anomalous regions of the image (Risholm et al., 2009, 2010; Nielsen et al., 2019), which may be accurate in situations such as cut tissue. However, throughout the majority of images, transformations are diffeomorphic, and we have chosen to work in the computational anatomy random orbit model to preserve properties that are useful for morphometry in addition to registration, such as the embedding of human anatomy into a metric space. Metamorphosis-based models (Miller et al., 2002; Li et al., 2011; Nithiananthan et al., 2012) allow image intensity to vary in certain regions to match anomalies, while mask-based models (Periaswamy and Farid, 2006; Sdika and Pelletier, 2009; Vidal et al., 2009; Chitphakdithai and Duncan, 2010b) or^9^ manually or automatically ignore these anomalies. Our method leverages the strengths of these last two, modeling both non-monotonic image intensity variation and masking in a generative statistical framework.

Our contribution to AD understanding stems from the need to bridge the gap between 3D imaging such as MRI, which can be obtained in living subjects over time, and 2D histopathology, which is the technique used to make the diagnosis postmortem. While some authors have successfully registered histology to MRI in well-controlled conditions (Adler et al., 2014), we believe that the generative model proposed here, which accommodates variable contrast and missing data, will be a valuable approach for handling typical data moving forward. This earlier work, which we compare to in our benchmarking results, is well appreciated and pioneers the space via the introduction of mutual information with the symmetric methods. However, because of the importance of the noise model in our setting for digital pathology, in which the target images contain many deletions of tissue and distortions within the image plane associated with extremely sparse collections, we have not focused on the symmetry of the orbit model described in (Christensen and Johnson, 2001; Avants et al., 2006, 2008). The statistical model for image formation of Equation (5) removes symmetry assumptions between template and target, such as are associated with other methods. This is because the correspondence of histology images to the atlas is an injection. The penalized Bayes estimator therefore computes deformations and variations only in the atlas image, as shown by the necessary maximizer condition of Equation (7a). This paper emphasizes the statistical estimation model in which the atlas is an idealized representation, i.e., the atlas is densely sampled to 100 microns isotropically, while the target has nonuniform sparse sampling to 1–2 mm with significant noise associated to nuisance dimensions that are unique to the histological preparation. An additional advantage of our statistical likelihood interpretation for estimating missing or distorted image data is that it allows us to perform Bayesian calculations such as are used in multi-atlas interpretation (Tang et al., 2012; Wang et al., 2012) and Bayesian segmentation (Tang et al., 2013).

This work advances the field of brain mapping in two important ways. First, it moves to exploit statistical models of image formation under the key assumption that the measured targets are sparse and noisy samples of the dense atlases. No symmetric property is available because mappings from histology slices to 3D atlases are injective: while there may be only one point in the atlas corresponding to each point in the target, the atlas is far more densely sampled. Second, this method accommodates mapping between images taking values in arbitrary dimensions in the presence of missing tissue and artifacts. This allows accurate brain mapping to expand from well-controlled clinical imaging to the massive diversity of neuroscience data. For example, in the mouse community, accurate image-mapping between Nissl-stained tissue and microscopy with multiple fluorophores is commonly required in the presence of variably dissected or damaged tissue. We are currently applying these techniques to CLARITY (Chung et al., 2013; Epp et al., 2015) and iDISCO (Renier et al., 2014) images in mouse and rat (Branch et al., 2018), serially sectioned mouse as part of the BRAIN Initiative Cell Census Network (Lee et al., 2018) and revisiting older datasets where images were excluded due to artifacts or damaged tissue.

## Data Availability Statement

The datasets analyzed and generated for this study can be found on the Center for Imaging Science's FTP server^10^. This repository contains four datasets and a README file that describes which datasets were used to make which figures. In particular, we include images from our phantom experiment (Figure 2), *ex-vivo* MRI images and their manual segmentations (Figures 1, 9, 11), low-resolution histology images that were used for registration (Figures 3, 4, 5, 7, 9, 11), and high-resolution histology images with detected tau tangle probability (Figures 10–12). Images from the benchmarking dataset can be acquired at^11^.

## Author Contributions

DT and MM designed the algorithm. DT wrote the resulting code and performed the experiments. TB performed the manual annotations. YK, JP, and JT prepared the histology images. ZH, JP, and SM prepared the post-mortem MRI. DT, SM, MA, JT, and MM conceived of this study and contributed to the writing and revising of the manuscript.

### Conflict of Interest

SM and MM own Anatomy Works, with SM serving as its CEO. This arrangement is being managed by Johns Hopkins University in accordance with its conflict of interest policies. The remaining authors declare that the research was conducted in the absence of any commercial or financial relationships that could be construed as a potential conflict of interest.

## Footnotes

^1^https://www.python.org

^2^https://www.tensorflow.org

^3^https://neurodata.io/reg

^4^https://github.com/Borda/BIRL

^5^https://anhir.grand-challenge.org/

^6^https://farid.berkeley.edu/downloads/code/qr_0.2.tar.gz

^7^http://www.mricloud.org

^8^www.scipy.org

^9^https://github.com/armaneshaghi/LesionFilling_example

^10^ftp://ftp.cis.jhu.edu/pub/data_share/tward_frontiers_neuroscience_2019/

^11^http://cmp.felk.cvut.cz/~borovji3/?page=dataset
