# Supplementary material for: Diffeomorphic Registration With Intensity Transformation and Missing Data: Application to 3D Digital Pathology of Alzheimer's Disease
Source: Front Neurosci. 2020 Feb 11;14:52. doi: 10.3389/fnins.2020.00052 (PMC7027169; doi:10.3389/fnins.2020.00052)
Supplement: Supplementary file 1 [file Data_Sheet_1.pdf]

DIFFEOMORPHIC REGISTRATION WITH INTENSITY TRANSFORMATION AND MISSING DATA: APPLICATION TO 3D DIGITAL PATHOLOGY OF ALZHEIMER'S DISEASE

APPENDICES

1 LDDMM CHAIN RULE AND AFFINE TRANSFORMATION PROOF

Equation (4a) is computed by application of the chain rule. First the derivative of E with respect to $F_\theta(I)$, second $F_\theta[I(\varphi^{-1})]$ is with respect to $I(\varphi^{-1})$, and third I with respect to the deformation field. The first and second steps are combined as

$$\frac{\partial E}{\partial[I(\varphi^{-1})]} = \frac{1}{\sigma^2}(F_\theta[I(\varphi^{-1})] - J) \cdot DF_\theta[I(\varphi^{-1})] \tag{8}$$

The third step is discussed in (Beg et al., 2005). Equation (4a) can be solved using a standard gradient descent approach.

The equation (4b) is the necessary condition with respect to the contrast. Since F_θ as a polynomial it is linear in θ, and so (4b) is a linear system solved exactly at each iteration of gradient descent.

Minimizing over both θ and transformation parameters means the result of registration will be independent of the family of transformations indexed by θ. An alternative approach is to minize over θ first, leading to an invariant cost:

$$C(I) = \min_\theta \frac{1}{2\sigma^2}\|F_\theta(I) - J\|^2_{L_2} \ .$$

Here is the proof that the affine transformation problem reduces to normalized cross correlation, which is invariant to affine transformations of the atlas image contrast.

COROLLARY (Unknown Affine Transformations Correspond to Normalized Cross Correlation Squared). *When F is an affine map from $\mathbb{R} \to \mathbb{R}$, our registration results will be invariant to affine transformations of our atlas, and* (4b) *can be solved analytically. For $N = M = 1$ and $a, b \in \mathbb{R}$, $F_{a,b}(t) = at + b$, minimizing $\frac{1}{2\sigma^2}\|aI \circ \varphi^{-1} + b - J\|^2_{L_2}$ is equivalent to maximizing the normalized cross correlation squared of $I \circ \varphi^{-1}$ with J.*

PROOF. For any fixed φ, optimal values of a, b can be found via a standard linear least squares estimation result, which gives $a = \mathrm{Cov}(I(\varphi^{-1}), J)/\mathrm{Var}(I(\varphi^{-1}))$ and $b = \bar{J} - a\overline{I(\varphi^{-1})}$ where $\bar{\cdot}$ corresponds to the expected value, and expectation, variance, and covariance are taken over all voxels in the images. Plugging these into squared-error cost gives

$$\begin{aligned}
&\frac{1}{2\sigma^2}\int_X \left| \frac{\mathrm{Cov}(I(\varphi^{-1}), J)}{\mathrm{Var}(I(\varphi^{-1}))} I(\varphi^{-1}(x)) + \bar{J} - \frac{\mathrm{Cov}(I(\varphi^{-1}), J)}{\mathrm{Var}(I(\varphi^{-1}))}\overline{I(\varphi^{-1})} - J(x)\right|^2 dx \\
&= -\frac{1}{\sigma^2}|X|\frac{\mathrm{Cov}^2(I(\varphi^{-1}), J)}{\mathrm{Var}(I(\varphi^{-1}))} + \frac{1}{2\sigma^2}|X|\,\mathrm{Var}(J) \\
&= -\frac{1}{\sigma^2}|X|\,\mathrm{Var}(J)\,\mathrm{NCC}^2(I(\varphi^{-1}), J) + \frac{1}{2\sigma^2}|X|\,\mathrm{Var}(J)
\end{aligned}$$

Up to constants that do not depend on the deformation, minimizing sum of square error with an unknown affine intensity transformation is equivalent to maximizing normalized cross correlation (NCC) squared.

2 PROOF THE MISSING DATA ALGORITHM IS AN EM ALGORITHM AND THEREFORE MONOTONIC IN INCOMPLETE DATA LOG-LIKELHOOD

We discretize the images via the lattice of sites forming a disjoint partition (voxels) $X = \cup_{i=1}^{S} \Delta x_i$. Each atlas type $a \in \mathcal{A} = \{a_1, a_2, \ldots,\}$ includes a deformation given by $\varphi_a = \int_0^1 v_t^a \circ \phi_t^a dt + id$, so the discrete values can be written as $I_i^a = \int_{\Delta x_i} I^a \circ \varphi_a^{-1}(y) dy$.

The observable random field $J_i, i = 1, \ldots, S$, is conditionally Gaussian with constant variances σ_a^2 and mean fields I_i^a determined by the atlas type $a \in \mathcal{A}$. In practice, we choose I^{a_1} as our atlas image, and I^{a_i} for $i \neq 1$ to be constant images. Since they are constant, we need not optimize over the deformations $v_t^{a_i}$ for $i > 0$. Augment the measured *incomplete-data* $\mathbf{Y} = \{J_i, i = 1, 2, \ldots, S\}$ with labels determining the atlas types $A_i \in \mathcal{A} = \{a_1, a_2, \ldots,\}$ generating the *complete-data* $\mathbf{X} = \{(J_i, A_i), i = 1, \ldots, S\}$. Model J_i as a Gaussian random variable with mean $I_i^{A_i}$ and variance $\sigma_{A_i}^2$. The complete-data penalized log-likelihood becomes:

$$\log f(\mathbf{X}; v) = \sum_{a \in \mathcal{A}} -\frac{1}{2\sigma^2} \int_0^1 \int_X A v_t^a \cdot v_t^a dx dt - \frac{1}{2\sigma_a^2} \sum_{i=1}^{S} \delta_a(A_i) |J_i - I_i^a|^2 \; .$$

The Kronecker-delta $\delta_a(\cdot)$ is 1 when the argument is a, and zero otherwise. The Expectation step (E-step) replaces these functions with their expected value, a posterior probability $\pi_a(i)$ at each voxel Δx_i.

THEOREM 1. *The Expectation Maximization algorithm is defined by the following at iteration (p):*

$$I^{a,(p)} = I^a \circ \varphi^{-1(p)}, a \in \mathcal{A},$$

E-step: $E\left(\log f(\mathbf{X}; v) | \mathbf{Y}, v^{a,p}\right)$

$$\pi_i^{a,(p+1)} = \frac{\frac{1}{\sqrt{2\pi\sigma_a^2}} \exp(-\frac{1}{2\sigma_a^2} |J_i - F_{\theta^{a,(p)}}(I_i^{a,(p)})|^2)}{\sum_{a' \in \mathcal{A}} \frac{1}{\sqrt{2\pi\sigma_{a'}^2}} \exp(-\frac{1}{2\sigma_{a'}^2} |J_i - F_{\theta^{a',(p)}}(I_i^{a',(p)})|^2)} \; , \; a \in \mathcal{A} \; ; \tag{9}$$

M-Step: $\arg\max E\left(\log f(\mathbf{X}; v) | \mathbf{Y}, v^{(p)}\right)$

$$v^{a,(p+1)} = \arg\max_{v^a} -\frac{1}{2\sigma^2} \int_0^1 \int_X A v_t^a \cdot v_t^a dx dt - \frac{1}{2\sigma_a^2} \sum_i \pi_i^{a,(p+1)} |J_i - F_{\theta^{a,(p)}}(I_i^{a,(p)})|^2$$

$$\theta^{a,(p+1)} = \arg\max_{\theta^a} -\frac{1}{2\sigma^2} \int_0^1 \int_X A v_t^{a,(p)} \cdot v_t^{a,(p)} dx dt - \frac{1}{2\sigma_a^2} \sum_i \pi_i^{a,(p+1)} |J_i - F_{\theta^a}(I_i^{a,(p)})|^2 \; .$$

Iterations $v^{a,(p+1)}, \theta^{a,(p+1)}$ with $\varphi^{a,(p+1)} = \int_0^1 v^{a,(p+1)} \circ \phi^{a,(p+1)} dt + id$ increase in incomplete-data log-likelihood.
