# Supplementary figures and images for: Diffeomorphic Registration With Intensity Transformation and Missing Data: Application to 3D Digital Pathology of Alzheimer's Disease
Source: Front Neurosci. 2020 Feb 11;14:52. doi: 10.3389/fnins.2020.00052 (PMC7027169; doi:10.3389/fnins.2020.00052)

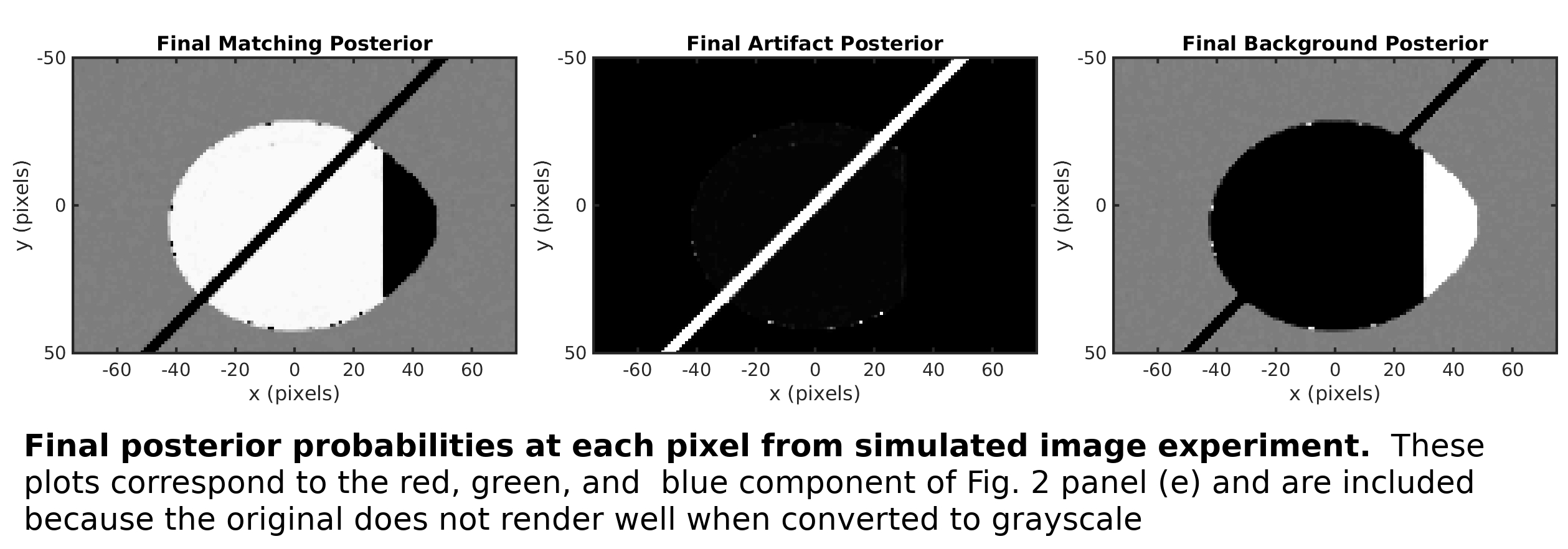

Supplement: Supplementary file 2 [file Image_1.JPEG]
